
JOURNAL INFORMATION
==============================
NLM Title Abbreviation: ScientificWorldJournal
Journal ID: ScientificWorldJournal
NLM Journal ID: 101131163
Journal ID: TSWJ

The Scientific World Journal

ISSN: 2356-6140
EISSN: 1537-744X
Publisher: Wiley

ARTICLE INFORMATION
==============================
PMCID: PMC6009702
PMID: 12806003
DOI: 10.1100/tsw.2002.213
Article ID: 896792
Article version: 1
Subjects: Founding Editorial

Founding Editorial – Bone Biology

Bronner Felix *
Department of BioStructure and Function, University of Connecticut Health Center, Farmington, CT, USA
*Felix Bronner: bronner@neuron.uchc.edu
Publication date: 2002
Electronic publication date: 2002 Mar 21
Volume: 2
First page: 787
Last page: 790
Received 2002 Mar 11; Accepted 2002 Mar 13
Copyright: Copyright © 2002 Felix Bronner.
Copyright year: 2002
License: This is an open access article distributed under the Creative Commons Attribution License, which permits unrestricted use, distribution, and reproduction in any medium, provided the original work is properly cited.
License URL: https://creativecommons.org/licenses/by/3.0/

Keywords: bone formation, bone resorption, osteoblasts, osteoclasts, osteocytes, hormones, signal molecules, parathyroid hormone, calcitonin, vitamin D, calcium metabolism, phosphate metabolism, osteoporosis, bone disorders, nutrition, aging, skeleton

==============================
Founding Editorial
*TheScientificWorldJOURNAL* (2002) 2, 787–790
ISSN 1537-744X; DOI 10.1100/tsw.2002.213

### Founding Editorial – Bone Biology

Felix Bronner
*Department of BioStructure and Function, University of Connecticut Health Center, Farmington, CT*

*Received March 11, 2002; Accepted March 13, 2002; Published March 21, 2002*

**KEY WORDS:** bone formation, bone resorption, osteoblasts, osteoclasts, osteocytes, hormones, signal molecules, parathyroid hormone, calcitonin, vitamin D, calcium metabolism, phosphate metabolism, osteoporosis, bone disorders, nutrition, aging, skeleton

**DOMAINS:** bone biology

The skeleton is a complicated vertebrate structure, comprised of bone cells that form, modulate, and resorb the extracellular structure of bone. It is the extracellular structure, made up of the bone mineral (largely calcium phosphate) and the bone matrix, which constitutes the visible skeleton and the mechanical support for the vertebrate body. The matrix is the protein structure on which the bone mineral is laid down, many components of which have been identified in recent years.

This simplified description of bone already implies the many scientific disciplines that have in recent decades begun to deal with the skeleton. Anatomists, histologists, inorganic and organic chemists, cell biologists, geneticists, pathologists, physiologists, and a variety of medical specialists — orthopaedists, endocrinologists, nephrologists, rheumatologists, gynecologists, to name just a few — all have an interest in how bone is formed and affected by the life cycle — growth, maturation, reproduction, aging — and in the now emerging abundance of actual and potential treatments of bone disorders and diseases.

It seems entirely appropriate, therefore, that the interdisciplinary, internet journal — *TheScientificWorldJOURNAL*, the journal without boundaries – has organized a Bone Biology Domain under the editorship of a wide variety of specialists. It is our hope and purpose to publish the results of first-rate research in this area. At the same time we intend to publish reviews, symposia in print, and analyses of controversial viewpoints or techniques, with the intent that new findings or re-evaluation of older findings can be viewed in the broad context of skeletal biology. Moreover, the networked arrangement that underlies *TheScientificWorldJOURNAL* and its editorial concept facilitates communication within and between disciplines. Understanding in the bone biology domain can therefore be viewed in an even wider perspective.

Osteoporosis — the change in bone mass and structure that occurs with aging in men and women — is a good example of a condition dealt with by many disciplines**.** Questions concerning the mechanisms regulating bone formation and resorption that cause the loss in bone mass are of interest to cellular and developmental biologists and to several of the medical specialists referred to above. Disturbances in the normally exquisite regulation of calcium homeostasis can play a role in disorders of bone metabolism, including osteoporosis. They therefore are another example

Email: Bronner@NEURON.UCHC.EDU
©2002 with author.

of the kind of problems that concern a broad spectrum of biological and medical specialists. Inasmuch as the development of a skeleton not subject to early fracture is also the result of an adequate calcium and protein intake, public health policy aimed at delaying the occurrence of osteoporosis must involve questions of nutrition policy and, if a change in food intake is desired, behavioral modification becomes an important issue. Similarly, if hormone replacement therapy is indicated to minimize the continued decrease in bone mass, the physiological and psychological consequences of such therapy must be explored.

With the increase in genetic knowledge questions concerning genetic aspects of osteoporosis are not only being asked, but are likely to receive answers. These answers will, it seems certain, involve not only individual genes, but arrays of genes that induce the synthesis of proteins that play a role in directing cells to their targets, that serve as signals inside cells and in cellular crosstalk. Also, assuring an adequate nutrient supply for the proper development of the skeleton may require, on a population basis, modification of agricultural and therefore economic policies.

As Principal Editor of the Bone Biology domain I have attempted to surround myself with colleagues as Associate Editors and members of the Editorial Board who are experts in the many aspects of the bone biology domain, and I am grateful for their willingness to provide the broad and deep knowledge needed for this task. The listing of their names and addresses contains also a self-description of their expertise. Authors of primary publications or of reviews are invited to submit their contributions to the Journal.

---

### EDITORIAL BOARD FOR
### BONE BIOLOGY

A domain of TheScientificWorld

### PRINCIPAL EDITOR

**Felix Bronner**
Department of BioStructure and Function
University of Connecticut Health Center, Farmington, CT, USA
*Research interests: Calcium metabolism (absorption, transport, homeostasis), nutrition, bone metabolism, vitamin D, parathyroid hormone, calcitonin*

### ASSOCIATE EDITORS

**Sylvia Christakos**
Department of Biochemistry and Molecular Biology, New Jersey Medical School, Newark, NJ, USA
*Research interests: endocrinology, Vitamin D - transcription and translation*

**Gerard Karsenty**
Department of Molecular and Human Genetics, Baylor College of Medicine, Houston, TX, USA
*Research interests: osteoblast differentiation, transcription, genetic pathways of bone remodeling and bone mineralization, calcitonin, leptin*

**T. Jack Martin**
St. Vincent's Institute of Medical Research, Melbourne, Australia
*Research interests: bone cell biology, calcium-regulating hormones, bone diseases of cancer, osteoporosis*

**Meinrad Peterlik**
Institute of Pathophysiology, University of Vienna Medical School, Vienna, Austria
*Research interests: pathophysiology, biochemistry, cell biology, cellular actions of vitamin D, calcium and phosphate homeostasis, cytokines and bone turnover, hormonal regulation of intestinal cell differentiation, pathogenesis and treatment of colon cancer, pathogenesis of osteoporosis*

**Carol C. Pilbeam**
Department of Medicine, University of Connecticut Health Center, Farmington, CT, USA
*Research interests: bone cell biology, cytokines and prostaglandins, mechanical loading of bone, bone loss of aging*

**Dwight Towler**
Director of the Division of Bone and Mineral Diseases, Washington University School of Medicine, St. Louis, MO, USA
*Research interests: biochemistry*

### EDITORIAL BOARD MEMBERS

**Mary C. Farach-Carson**
Department of Biological Sciences, University of Delaware, Newark, DE, USA
*Research interests: endocrinology, calcitropic hormones, osteoblasts*

**Francis Glorieux**
Genetics Unit, Shriner's Hospital for Children, Montreal, QC, Canada
*Research interests: musculoskeletal genetic research*

**Stephen M. Krane**
Center for Immunology and Inflammatory Diseases, Harvard Medical School, Boston, MA, USA
*Research interests: bone pathophysiology, sugar transport, bone turnover and controls of synthesis and degradation of the extracellular matrix, rheumatic and metabolic bone diseases, parathyroid hormone and calcitonin, collagenase, collagen synthesis and degradation in genetic and acquired diseases, inflammatory mechanisms in joint diseases*

**Barbara E. Kream**
Department of Medicine, University of Connecticut Health Center, Farmington, CT, USA
*Research interests: bone cell biology, cytokines, parathyroid hormone*

**J. Edward Puzas**
University of Rochester School of Medicine, Rochester, NY, USA
*Research interests: bone cell biology*

**A. Hari Reddi**
Center for Tissue Regeneration and Repair, University of California–Davis, Davis, CA, USA
*Research interests: cartilage, transformation of bone cells to calcified bone*

**Justin Silver**
Mineral Metabolism Unit, Hadassah University Hospital, Jerusalem, Israel
*Research interests: endocrinology, nephrology, vitamin D, parathyroid hormone*

**Harriet Tenenhouse**
Department of Pediatrics and Human Genetics, McGill University, Montreal, QC, Canada
*Research interests: molecular basis of inherited rickets, renal stone formation in humans, use of mouse models to study pathophysiology*

**This article should be referenced as follows:**

Bronner, F. (2002) Founding editorial – Bone Biology. *TheScientificWorldJOURNAL* **2**, 787–790.
